# Supplementary material for: An evaluation of the impact of social and structural determinants of health on forgone care during the COVID-19 pandemic in Baltimore, Maryland
Source: PLoS One. 2024 May 13;19(5):e0302064. doi: 10.1371/journal.pone.0302064 (PMC11090349; doi:10.1371/journal.pone.0302064)
Supplement: S1 File — (DOCX) [file pone.0302064.s001.docx]

**S1. Parent Study Survey Questions**

**C-Forward Parent Survey Questions**

DEMOGRAPHICS

1. What was your biological sex assigned at birth?

- Male
- Female
- Non-binary
- None of these describe me
- Prefer not to answer

1. What terms best express how you describe your gender identity?

- Male/Man
- Female/Woman
- Non-binary
- Transgender man/Female-to-Male (FTM)
- Transgender woman/Male-to-Female (MTF)
- Gender non-binary/Genderqueer/Gender nonconforming
- Agender
- Bigender
- None of these describe me
- Prefer not to answer

1. Which of the following best describe your marital status?

- Single
- Monogamous, in a relationship
- In more than one relationship
- Engaged
- Married
- Other (Specify_____________________________)

1. What is your highest level of education you have achieved outside or in the United States? Grades are roughly equivalent to years of school.

- Have never gone to school
- 5^th^ grade or less
- 6^th^ to 8^th^ grade
- 9^th^ to 12^th^ grade, no diploma
- High school graduate or GED completed
- Some college level/technical/vocational/associate’s degree
- Bachelor’s degree
- Other advanced degree (Master’s, doctoral degree)
- Prefer not to answer
- Don’t know

1. We would like to know about what you do- Are you working now, looking for work, retired, keeping house, a student or what? ***CHECK ALL THAT APPLY***

- Working full-time (40 hours per week)
- Working part-time (Less than 40 hours per week)
- Only temporarily laid off, sick leave or maternity leave
- Looking for work, unemployed
- Retired
- Disabled, permanently or temporarily
- Keeping house
- Student
- Other (Specify: ___________________________)
- Prefer not to say
- Don’t Know

1. Are you considered an essential worker? An essential worker is someone who was required to go to work even when stay at home orders were in place in Maryland.

- Yes
- No
- Don’t Know

1. Would any of these describe where you work?

- Nursing care facilities
- Visiting nurse or home health aide service
- Building cleaning services
- Public transportation
- Corrections facility
- EMT or paramedic services
- Meat packing farm facility
- Agriculture and food production facility
- Grocery Store
- Construction
- No

1. Are you currently a caregiver for a dependent/dependents in your home? A dependent is anyone who relies on you for help with activities of daily living, including children under the age of 18 years or an older adult with chronic disease or disability.

- Yes
- No
- Don’t Know

1. What was your household annual income in 2019 from all sources before taxes? This includes all income from both formal and informal employment.

- less than $15,000
- $15,000 - $19,999
- $20,000 – $24,999
- $25,000 - $34,999
- $35,000 - $49,999
- $50,000 - $74,999
- $75,000 - $99,999
- $100,000 and above
- Don’t know
- Refuse to Answer

1. Including yourself, how many people depend on this income? This can include people who don’t live with you now.

- 1
- 2
- 3
- 4
- 5
- 6
- 7
- 8
- 9
- 10 or more
- Don’t know
- Refuse to answer

1. Prior to the COVID-19 pandemic (March 1, 2020), was your household paying lower rent because the Federal, State or Local government was paying part of the cost?

- Yes
- No
- Don’t Know
- Refuse to Answer

1. Were you born in the United States?

- Yes
- No
- Don’t Know
- Refuse to Answer

1. Do you speak a language other than English at home?

- Yes
- No (***GO TO Q14***)

13a. What Language(s): **CHECK ALL THAT APPLY**

- Spanish
- Vietnamese
- Mandarin
- Cantonese
- Tagalog
- Hawaiian
- Ilokano
- Navajo
- Other (Specify:___________)

1. Which of the following best represents how you think of yourself at this time?

- Straight; that is, not gay or lesbian, etc.
- Gay
- Lesbian
- Bisexual
- None of these describe me
- Prefer not to answer

COMORBIDITIES AND HEALTH CARE ACCESS

*The next questions are about health conditions you may have and how your health care has been impacted by the COVID-19 pandemic.*

1. Are you currently pregnant?

- Yes
- No
- Not applicable

1. How tall are you without shoes? *Please choose the units you would like to use for height.*

*Feet* *Inches*

*Meters* *Centimeters*

1. How much do you weigh without clothes or shoes? *Please choose the units you would like to use for weight.*

*If you are currently pregnant, how much did you weigh before your pregnancy?*

*Pounds*

*Kilograms*

1. Do you have any of the following health conditions? ***CHECK ALL THAT APPLY***

|  | Yes | No | Don’t Know/ Unsure |
| --- | --- | --- | --- |
| 1. Diabetes | ⬜ | ⬜ | ⬜ |
| 1. Cardiovascular disease (CVD or heart disease) | ⬜ | ⬜ | ⬜ |
| 1. Hypertension (HTN, high blood pressure) | ⬜ | ⬜ | ⬜ |
| 1. Immunocompromised condition | ⬜ | ⬜ | ⬜ |
| 1. Autoimmune disease | ⬜ | ⬜ | ⬜ |
| 1. Cancer diagnosis and/or treatment within past 12 months | ⬜ | ⬜ | ⬜ |
| 1. Chronic kidney disease | ⬜ | ⬜ | ⬜ |
| 1. Asthma | ⬜ | ⬜ | ⬜ |
| 1. Chronic obstructive pulmonary disease (COPD) | ⬜ | ⬜ | ⬜ |
| 1. Other chronic lung disease | ⬜ | ⬜ | ⬜ |
| 1. Sickle cell anemia | ⬜ | ⬜ | ⬜ |
| 1. Depression | ⬜ | ⬜ | ⬜ |
| 1. Alcohol or substance use disorder | ⬜ | ⬜ | ⬜ |
| 1. Injection drug use | ⬜ | ⬜ | ⬜ |
| 1. Other mental health disorder | ⬜ | ⬜ | ⬜ |
| 1. Other chronic condition | ⬜ | ⬜ | ⬜ |

4a1. If you said YES to Cardiovascular disease (***Q4b***), which of the following cardiovascular conditions do you have?

|  | Yes | No |
| --- | --- | --- |
| 1. Congestive heart failure | ⬜ | ⬜ |
| 1. Myocarditis/pericarditis | ⬜ | ⬜ |
| 1. History of stroke(s) | ⬜ | ⬜ |
| 1. History of heart attack(s) | ⬜ | ⬜ |
| 1. Other | ⬜ | ⬜ |

Describe: ________________________________________________________________

4a2. If you said YES to Immunocompromised condition (***Q4d***), which of the following immune disorders do you have?

|  | Yes | No |
| --- | --- | --- |
| 1. Transplant recipient | ⬜ | ⬜ |
| 1. HIV positive | ⬜ | ⬜ |
| 1. Other | ⬜ | ⬜ |

Describe: ________________________________________________________________

1. Do you take chronic medication for any of the conditions listed above?

- Yes
- No *(****GO TO Q6****)*
- Don’t know *(****GO TO Q6****)*

5a. Since the COVID-19 pandemic, (March 1, 2020), have you experienced any delays or interruptions of treatment as a result of COVID-19?

- Yes
- No
- I’m not sure/Don’t know

1. Since the COVID-19 pandemic (March 1, 2020), did you…

|  | Yes | No | Don’t Know/ Unsure |
| --- | --- | --- | --- |
| 1. Need to see a health care provider for a routine follow-up visit or a new health or mental health concern that was not related to COVID-19 |  |  |  |
| 1. Skip/miss any in person or video/phone appointments with a health care provider because of COVID-19 |  |  |  |
| 1. Go to any appointment with a health care provider in person |  |  |  |
| 1. Have an appointment with a health care provider by phone or video |  |  |  |

1. Since the COVID-19 pandemic (March 1, 2020), did you need to go to the emergency room/hospital for a condition that was not related to COVID-19?

- Yes
- No *(****GO TO Q8****)*
- Don’t Know *(****GO TO Q8****)*

7a. Did you go to the emergency room/hospital when you needed to?

- Yes
- No
- Don’t Know

1. Since the COVID-19 pandemic (March 1, 2020), did you need to have a major medical or dental procedure such as surgery?

- Yes
- No *(****GO TO Q9****)*
- Don’t Know *(****GO TO Q9****)*

8a. Did you postpone this medical or dental procedure because of COVID-19?

- Yes
- No
- Don’t Know

8b. Have you now had this medical or dental procedure?

- Yes
- No
- Don’t Know

1. Would you say your health in general is excellent, very good, good, fair, or poor?

- Excellent
- Very good
- Good
- Fair
- Poor
- Prefer not to answer
- Don’t know

1. The next questions are about limitations that you might have.

|  | Yes | No | Prefer not to say |
| --- | --- | --- | --- |
| 1. Do you have a disability that interferes with your ability to carry out daily activities? Examples of daily activities include walking, climbing stairs, shopping, balancing a checkbook, bathing or dressing. |  |  |  |
| 1. Are you limited in any way in any activities because of physical, mental, or emotional problems |  |  |  |
| 1. Do you have serious difficulty concentrating, remembering things, or making decisions |  |  |  |
| 1. Do you have difficulty doing errands alone, such as visiting a doctor’s office or shopping |  |  |  |
| 1. Do you now have any health problem that requires you to use special equipment, such as a cane, a wheelchair, a special bed, or a special telephone (*Include occasional use or use in certain circumstances)* |  |  |  |
| 1. Do you have serious difficulty walking or climbing stairs |  |  |  |
| 1. Are you blind or do you have serious difficulty seeing even when wearing glasses |  |  |  |
| 1. Do you have difficulty dressing or bathing |  |  |  |

1. Are you deaf or do you have serious difficulty hearing?

- Yes
- No *(****GO TO SECTION 5****)*
- Prefer not to say

*(****GO TO SECTION 5****)*

11a. Do you wear a hearing aid or have a cochlear implant?

- Yes
- No
- Prefer not to say

CORONAVIRUS IMPACT AND PANDEMIC STRESS

1. At any time since March 1, 2020,

|  | No | Yes | Not applicable | Don’t Know/Unsure |
| --- | --- | --- | --- | --- |
| 1. Have you or anyone in your household lost housing or become homeless |  |  |  |  |
| 1. Has your household been unable to pay your rent/mortgage |  |  |  |  |

**CoVPN 5002 Parent Survey Questions**

DEMOGRAPHICS

*Interviewer (read aloud): First, we would like to ask you some questions about you and your household.*

1. How old are you? (For participants less than 1 years old enter “0” and then enter age in months below.)___________
   1. Infant age in months___
2. What is your ethnicity? (*mark one)*

- Hispanic
- Non-Hispanic
- Don’t Know/ Not sure
- Prefer not to answer

1. What is your race? (*Mark all that apply)*

- American Indian or Alaskan Native
- Asian
- Black or African American
- Native Hawaiian or Other Pacific Islander
- White or Caucasian
- Other
- Don’t Know/ Not sure
- Prefer not to answer

1. Are you currently a full time or part time student? (13 and older only)

- Yes – full time
- Yes – Part time
- No

1. What is the highest level of education you have completed?

- No formal education
- Pre-kindergarten
- Kindergarten
- Elementary school (1st to 5^th^ grade)
- Middle school (6^th^ – 8^th^ grade)
- High school diploma/GED
- Some college/university
- College/university degree
- Post-graduate degree

1. What is your marital status? (13 and older only)

- Single
- Married or Cohabitating
- Widowed
- Divorced
- Separated

1. What is the zip code of your primary residence? ______________
2. What best describes your current primary residence?

- Live in a house or apartment
- Live in a nursing home or retirement home *(Skip to Q10)*
- Live in a hotel or motel *(Skip to Q10)*
- Live in a drug recovery or transitional house *(Skip to Q10)*
- Live in a homeless shelter *(Skip to Q10)*
- Prefer not to answer *(Skip to Q10)*
- Other *(Skip to Q10)*

1. How many bedrooms are in the residence? ________
2. Do you live alone, with a group, family, or multiple families?

- Alone *(Skip to Q12)*
- Group (small, residential facilities located within a community, recovery residence, transitional housing, or roommates)
- Single family (parents/guardians, children, grandparents, etc.)
- Multiple families (two or more families)

1. How many people are currently living in your household, including yourself? _______
2. Are you a caregiver to anyone in your family or household? (13 and older only)

- Yes
- No
- Don’t Know/Not Sure

1. What best describes your current employment status? (13 and older only)

- Employed – full time
- Employed – part time
- Working – not formal employment
- Not currently employed *(Skip to Q16)*
- Retired *(Skip to Q16)*
- Disabled (not currently employed due to long- or short-term disability) *(Skip to Q16)*
- Other: ____________

1. Are you an essential worker? (Essential workers are exempt from stay at home and shelter in place orders and must report to their place of work. Essential workers include but are not limited to those working in public health/health care, law enforcement, public safety, first responders, food and agriculture, energy and electricity, petroleum, water and wastewater, transportation, public works, communications and IT, and others.) (13 and older only)

- Yes
- No *(Skip to Q16)*
- Prefer not to answer *(Skip to Q16)*

1. Since March 2020, have you experienced any discrimination (such as being treated badly, harassed, threatened, isolated) from anyone because you are an essential worker? (13 and older only)

- Yes
- No
- Don’t Know/ Not sure

*Interviewer (read aloud): The next question is about your sex. When I ask about your sex, I am asking about what sex you were determined to be at birth. When babies are born, they are generally labeled by someone as “male” or “female” based on their genitals (sex organs)*

1. What was your sex assigned at birth? (check one)

- Male
- Female

*Interviewer (read aloud): The next question asks about gender. Gender is the social part of being male or female. It relates to your self-identity. When I ask about gender, I am asking about whether you regard yourself to be male, female, gender non-conforming transgender male, transgender female, or if you identify yourself in an additional category.*

1. What is your current gender identity? *(check one)*

- Male
- Female
- Gender non-conforming
- Transgender female
- Transgender male
- Additional category, specify ___________
- Prefer not to answer

1. What is your sexual orientation? *(mark all that apply)* (13 and older only)

- Gay/Lesbian/Homosexual
- Bisexual
- Queer
- Two-Spirit
- Straight/Heterosexual
- Additional Category, Please Specify _______________________________
- Don’t know/ Not sure
- Prefer not to answer

1. What was your total household income in the past 12 months?

*Note to interviewer: if a person lives with multiple people (e.g. roommate(s)) and does not know the income of everyone else, instruct them to respond with their total personal income.*

- <$15,000
- $15,000 - $24,999
- $25,000 - $34,999
- $35,000 - $49,999
- $50,000 – $74,999
- $75,000 - $99,999
- $100, 000 - $149,999
- $150,000 - $199,999
- >$200,000
- Don’t Know/Not sure
- Prefer not to answer

MEDICAL HISTORY

*Interviewer (read aloud): We are now going to ask about your medical conditions and how they may have been affected by the COVID-19 pandemic. Please answer these questions to the best of your ability.*

1. Have you ever been diagnosed by a healthcare professional with any of the following medical conditions? Pick all that apply

| Asthma | Yes | No |
| --- | --- | --- |
| Other chronic lung disease | Yes | No |
| Heart disease | Yes | No |
| Hypertension (high blood pressure) | Yes | No |
| Cancer chemotherapy in the last 12 months | Yes | No |
| Other immunosuppressive condition | Yes | No |
| HIV | Yes | No |
| Diabetes | Yes | No |
| Kidney or renal disease | Yes | No |
| Liver Disease | Yes | No |
| Sickle cell disease | Yes | No |
| Obesity | Yes | No |
| Mental health condition | Yes | No |
| Substance use disorder | Yes | No |
| Other chronic medical conditions that have not already been mentioned | Yes | No |

1. Are you limited in any way in your daily activities because of a physical, mental, or emotional disability? (Check all that apply)

- Physical disability
- Mental disability
- Emotional disability
- None of the above
- Prefer not to answer

1. Do you have any medical conditions that require medication or routine visits to the doctor?

- Yes
- No *(Skip to next section)*

1. Because of the COVID-19 pandemic, were you unable or unwilling to attend scheduled appointments with your health care provider?

- Yes, I did miss appointments
- No, I did not miss an appointment *(Skip to Q6)*
- No, I did not have any appointments to miss *(Skip to Q6)*
- Don’t Know/ Not sure *(Skip to Q6)*

1. What is the main reason you missed these appointments with your healthcare provider?

- My clinic cancelled my appointment because of COVID-19
- I had symptoms of COVID-19 so did not go
- I felt good, I did not need to go
- I did not have money or insurance
- Inconvenient (location/hours/times, etc.)
- Forgot to go/missed appointment
- Disrespected by the office or medical staff
- I could not attend telemedicine visit
- Did not have transportation
- I postponed my appointment until it was safe to go
- Other

1. Because of the COVID-19 pandemic, were you unable to take any of your medications?

- Yes, I could not get a refill from the pharmacy
- Yes, I did not get my refill from the pharmacy because I was concerned about social distancing, interacting with others, or exposure to coronavirus
- Yes, I have medication in my possession, but I forgot to take them
- Yes, I could no longer afford them because of lost employment or insurance.
- No
- Don’t Know
- Prefer not to answer

CoVPN 5002 Sub-Survey (additional survey questions taken by participants at the Baltimore, MD research site who enrolled starting in April 2021)

1. Since the COVID-19 pandemic (March 1, 2020), did you need to see a health care provider for a routine follow-up visit or a new health or mental health concern that was not related to COVID-19?
   1. Yes
   2. No
   3. Don’t know
2. Since the COVID-19 pandemic, (March 1, 2020), have you experienced any difficulties or delays in obtaining health care as a result of COVID-19?
   1. Yes
   2. No
   3. Don’t know
   4. Not applicable
3. Since the COVID-19 pandemic, did you have an appointment with a health care provider by phone or video?
   1. Yes
   2. No
   3. Don’t know
4. Since the COVID-19 pandemic (March 1, 2020), did you need to go to the emergency room/hospital for a condition that was not related to COVID-19?
   1. Yes
   2. No (*Skip to Q8*)
   3. Don’t know *(Skip to Q8*)
5. Did you go to the emergency room/hospital when you needed to?
   1. Yes
   2. No
   3. Don’t know
6. Since the COVID-19 pandemic (March 1, 2020), did you need to have a major medical or dental procedure such as a surgery?
   1. Yes
   2. No *(skip to Q11)*
   3. Don’t know *(skip to Q11)*
7. Did you postpone this medical or dental procedure because of COVID-19?
   1. Yes
   2. No *(skip to Q11)*
   3. Don’t know *(skip to Q11)*
8. Have you now had this medical or dental procedure?
   1. Yes
   2. No
   3. Don’t know

COVID-19 IMPACT

*Interviewer (read aloud): We would like to ask you about the impact COVID-19 has had on your daily life, your household, and how you feel. If no experiences apply to you, please respond with not applicable. Some of these questions may be very personal. Please be assured that your answers will remain completely confidential.*

1. Did you experience any of the following because of the COVID-19 pandemic?

| Financial | | | |
| --- | --- | --- | --- |
| Worrying about paying rent/mortgage | Yes | No | N/A |
| Loss of housing | Yes | No | N/A |
